# Elevated procalcitonin levels in patients with acetaminophen intoxication: two case reports: A CARE-compliant article

**DOI:** 10.1097/MD.0000000000018882

**Published:** 2020-02-14

**Authors:** Jung Hwan Ahn, Young Suk Cho, Gyu Chong Cho

**Affiliations:** a Department of Emergency Medicine, Ajou University School of Medicine, Suwon; b Department of Emergency Medicine, Kangdong Sacred Heart Hospital, Hallym University School of Medicine, Seoul, Republic of Korea.

**Keywords:** acetaminophen, acute liver failure, intoxication, paracetamol, procalcitonin

## Abstract

**Rationale:**

Procalcitonin (PCT) is used as a biomarker for identifying the occurrence of sepsis. Previous studies have reported high levels of PCT with acetaminophen intoxication without evidence of infection. Here, we report two patients with acetaminophen intoxication with high levels of PCT without showing any symptoms of bacterial infection.

**Patient concerns:**

This case study examined two unrelated patients with acetaminophen intoxication admitted to emergency at different times. The first patient was admitted to the emergency department after ingesting approximately 8000 mg (153.8 mg/kg) of acetaminophen. On admission, C-reactive protein (CRP), glutamic oxaloacetic transaminase (GOT), and glutamic pyruvic transaminase (GPT) were normal. PCT and acetaminophen levels were 31.89 ng/mL and below 0.5 μg/mL, respectively. The second patient was admitted to the emergency department 8 h after ingesting ∼23,600 mg (280.6 mg/kg) of acetaminophen. By the second day of admission, GOT and GPT increased to 2508 and 1473 IU/L, respectively. PCT was 45.66 ng/mL with acetaminophen level at 116.9 μg/mL. Both patients were clear of symptoms associated with bacterial infection.

**Diagnosis:**

Acetaminophen intoxication.

**Interventions:**

N-acetylcysteine was given intravenously to both patients for 20 h per protocol.

**Outcomes:**

Both patients were discharged without complications.

**Lessons:**

Observations suggests that elevated levels of PCT in patients intoxicated with acetaminophen may be associated with involvement of other organs impacted by cytokine stimuli from sterile inflammation resulting from hepatic damage rather than PCT secretion directly caused by hepatic cell damage.

## 1 Introduction

Procalcitonin (PCT) is used as an effective biomarker in identifying sepsis as well as to help determine the duration of antibiotics used in a patient.^[1–5]^ However, recent studies have shown that bacterial infection may not be the only cause resulting in elevated PCT levels. PCT levels can be elevated in patients experiencing acute liver failure (ALF) associated with acetaminophen intoxication without infection.^[5–7]^ This demonstrates that a measure of PCT does not always correlate with the probability of infection.^[5–7]^ One study has documented extremely elevated levels of PCT in children with ALF due to acetaminophen intoxication without evidence of bacterial infection that could not be explained by drug induced liver cell injury and/or hidden bacterial infection.^[5]^ Although the exact mechanisms of increased PCT levels in acetaminophen intoxication were unclear, the results of these previous studies suggest that high PCT levels in acetaminophen intoxication might be associated with involvement of other organs affected by cytokine stimuli in addition to liver cell damage.^[5–7]^

In this case report, we experienced two patients admitted to the emergency department with acetaminophen intoxication. Both patients had high levels of PCT without showing any symptoms of bacterial infection. While one patient showed elevated level of liver enzymes, the second patient had normal levels. However, PCT levels in both patients were much higher than that of a patient diagnosed with sepsis. Our observations documented in this case report suggests that other mechanisms such as involvement of other organs impacted by cytokine stimuli other than drug induced liver cell damage might contribute in elevating levels of PCT in patients with acetaminophen intoxication.

## 2 Case report

### 2.1 Case 1

A 24-year-old woman was admitted to the emergency department after ingesting ∼8000 mg (153.8 mg/kg) of acetaminophen in 12 h. She arrived at the emergency room after closely spaced ingestion of 4 tablets every 3 h for a total of 16 tablets from 08:00 to 22:00. On admission, the patient was mentally alert, although she suffered from epigastric pain, nausea, and vomiting. Vital signs upon arrival at our emergency department were as follows: blood pressure, 110/80 mm Hg; pulse rate, 66 times/min; respiratory rate, 20 times/min; and body temperature, 37.0°C. The patient reported direct tenderness around the epigastric area. However, she did not show signs of rebound tenderness or costovertebral angle tenderness.

N-acetylcysteine was administrated for 20 h, ∼13 h after the first intake of acetaminophen by the patient. C-reactive protein (CRP), glutamic oxaloacetic transaminase (GOT), glutamic pyruvic transaminase (GPT), and white blood cell (WBC) counts were normal. However, PCT was measured at 31.89 ng/mL at ∼60 h after the first intake of acetaminophen by the patient. Acetaminophen drug level was <5.0 μg/mL at ∼27 h after the first intake of acetaminophen. All laboratory results are summarized in Table 1. An ultrasound did not reveal any abnormal hepatic parenchymal echogenicity or bile duct dilatation. There were no indications suggesting the probability of infection such as rigor or fever. The patient was discharged from the hospital without complications four days after she was first admitted to the emergency department.

**Table 1:**
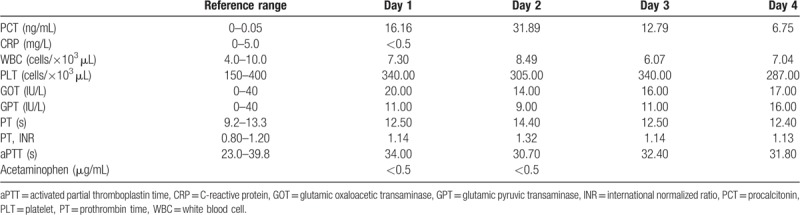
Results of laboratory findings for case 1.

### 2.2 Case 2

A 51-year-old man was admitted to emergency department at 8 h after ingesting 23,600 mg of acetaminophen (279.6 mg/kg). This patient was also mentally alert on admission. He explained that he felt generally weak without any discomfort. Vital signs on arrival in the emergency department were as follows: blood pressure, 100/60 mm Hg; pulse rate, 84 times/min; respiratory rate, 20 times/min; and body temperature, 36.0°C. A physical examination of the patient did not reveal anything unusual or anything out of the ordinary. N-acetylcysteine was also administered for 20 h ∼8 h after the first intake of acetaminophen by the patient. CRP, GOT, GPT, and WBC count were also normal.

By the second day of admission, GOT and GPT increased to 2508 and 1473 IU/L, respectively. Approximately at 56 h after first intake of acetaminophen, PCT level was elevated to 45.66 ng/mL. Acetaminophen drug level was 116.9 μg/mL. On the third day of admission, GOT and GPT decreased to 1382 and 1370 IU/L, respectively. Laboratory findings are summarized in Table 2. An ultrasound revealed a mild degree of fatty liver with hepatomegaly without bile duct dilatation. This patient did not show any signs associated with infection such as rigor or fever during his entire stay at the hospital. On the sixth day of admission, GOT and GPT was 53 and 357 IU/L, respectively. The patient was discharged since he did not show any unusual symptoms in addition to the daily decrease of his liver enzyme levels. He revisited to the out-patient department of gastroenterology the next day for a follow-up examination. There were no unusual symptoms. His liver enzyme returned to normal levels (GOT at 32 IU/L and GPT at 31 IU/L). The results of the laboratory tests performed in the out-patient department of gastroenterology are also summarized in Table 2.

**Table 2:**
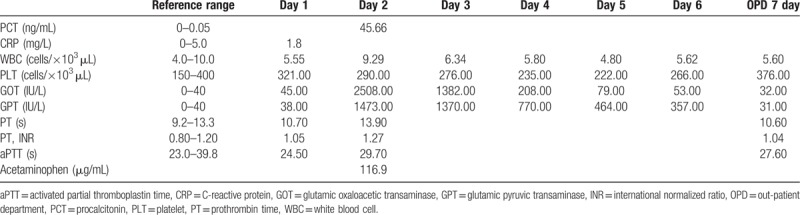
Laboratory results for case 2.

Informed consent was obtained for the current case report. Approval (IRB no. 2019-03-010) from the Institutional Review Board of Kangdong Sacred Heart Hospital, Hallym University School of Medicine, Seoul, Republic of Korea, was obtained.

## 3 Discussion

PCT is a precursor of calcitonin.^[1,3–6]^ It is secreted by thyroid C-cells and undetectable when metabolism is normal.^[3]^ Although the underlying mechanisms in PCT secretion are still unclear, several studies have reported that PCT is also secreted by and/or produced in the liver, testis, lung, prostate, kidney, small intestine, brain, heart, pancreas, thymus, and urinary bladder among other organs.^[3,8]^ Although infection is the most common cause of PCT level elevation, other reported causes include severe and prolonged cardiogenic shock, heat shock, trauma, severe pancreatitis, rhabdomyolysis, certain types of autoimmune disorders, lung cancer with liver metastasis, hepatocarcinoma with increased tumor size and poor prognosis, liver transplantation in children, and severe renal and liver dysfunction.^[4,7,9–12]^

High PCT levels have been reported in patients with ALF due to acetaminophen intoxication without infection.^[5,6]^ However, to the best of our knowledge, the underlying mechanisms are still unknown. It is also unclear whether there is an association among PCT, blood acetaminophen, and liver enzyme (GPT or GOT) levels. In most studies, there was no detailed description of the pathogenesis of PCT level elevation and the association between PCT and GPT levels in cases of acetaminophen intoxication. Tschiedel et al^[5]^ reported the correlation coefficient of PCT and acetaminophen levels at −0.45 (*P* = .15).^[5]^ Based on this value, the correlation between PCT and acetaminophen levels was not statistically significant. However, it is interesting to note that PCT levels were higher in patients with detectable blood acetaminophen levels than in those with undetectable blood acetaminophen levels, regardless of intoxication.^[5]^ Moreover, in the study by Tschiedel et al,^[5]^ the correlation between PCT and GPT, GOT, INR, and LDH levels was analyzed, and the results were as follows: GPT (*r* = 0.66, *P* = .02), GOT (*r* = 0.67, *P* = .02), INR (*r* = 0.53, *P* = .08), and LDH (*r* = 0.63, *P* = .03).^[5]^ According to this study, there is a mild-to-moderate positive correlation between PCT and GPT levels.^[5]^ Tschiedel et al^[5]^ proposed that there are two possible reasons for elevated PCT levels in patients with acetaminophen intoxication, other than acetaminophen-induced liver cell damage. First, when acetaminophen-induced liver damage occurs, damaged hepatocytes can result in damage-associated molecular patterns that can induce inflammasomes and inflammation in the liver.^[13]^ Sterile inflammation can be induced by cytokines IL-1 beta and tumor necrosis factor alpha.^[13]^ While it appears that more research is required to clarify all sources of PCT, it is evident that cytokine stimuli such as interleukin 2 and tumor necrosis factor alpha can act as mediators to elevate PCT levels.^[10,14]^ These cytokines after acetaminophen induced liver cell damage might partially explain the increase in PCT in patients with acetaminophen intoxication.^[5]^ The second reason was that there might be an association between PCT increase and the role of other organs such as endothelial cell.^[5,15]^ One case report has demonstrated the association between amphetamine intoxication and elevated PCT levels.^[15]^ This report might suggest that an increase in PCT levels may be associated with PCT secretions in organs with tissue damage or in immune cells. In case 2 of our study, the blood acetaminophen level was 116.9 μg/mL, and the GPT level was 1473 IU/L. The PCT level was 45.6 ng/mL, a level much higher than that in patients suspected of having sepsis^[2,7,12]^ or liver cell damage.^[9–11]^ These results are similar to those of previous studies.^[5,6]^ However, it is interesting to note that in case 1 of our study, the blood acetaminophen level was <5.0 μg/mL and the GPT level was 16 IU/L, but the PCT level was 31.9 ng/mL, a level much higher than that in patients suspected of having sepsis^[2,7,12]^ or liver cell damage.^[9–11]^ Previous studies have reported a positive correlation between blood acetaminophen and GPT levels.^[13,16–21]^ The authors of this study would like to emphasize that case 1 showed repeated supratherapeutic ingestion (staggered overdose ingestion), where the liver enzyme levels were normal and acetaminophen levels were detectable but not very high. Case 1, as staggered overdose ingestion, was different from single intoxication cases that have been reported in most previous studies and case 2 in this case report. Staggered overdose ingestions results in lower survival rates and higher rates of complications, such as hepatic encephalopathy, coagulopathy, electrolyte imbalance, and thrombocytopenia than single-dose acetaminophen intoxication.^[18]^ Moreover, in this study, while the doses of acetaminophen between single-dose acetaminophen intoxication and staggered overdose ingestion were similar, the blood acetaminophen and liver enzyme (GPT) levels were significantly lower in the staggered overdose case.^[18]^ Another study suggested that patients in the staggered overdose ingestion group with GPT/GOT levels <50 IU/L are unlikely to have hepatotoxicity.^[19]^ This study also reported that in patients receiving acetaminophen with GOT/GPT levels <50 UI/L, the mean ingested dose of acetaminophen was 10.6 (95% confidence interval, 9.4–11.7) g/day and the mean acetaminophen level at presentation was 27 mg/L (95% confidence interval, 20–34 mg/L).^[19]^ Although we can only reference one study, the patient in case 1 who was admitted for staggered overdose ingestion falls in the category of patients with a GPT level <50 IU/L, as categorized by Frank et al.^[19]^

Hepatotoxicity due to acetaminophen is caused by N-acetyl-p-benzoquinonimine (NAPQI), which is formed by oxidative metabolism and is mediated by cytochrome P450 enzymes (primarily CYP2E1).^[16,20,21]^ A study, although performed on rats, found indirect evidence that proved CYP2E1 activity was induced in cases of acetaminophen ingestion below hepatotoxicity doses, even though liver enzyme levels were not elevated.^[20]^ This study proposed that hepatotoxicity may develop in low acetaminophen doses with normal liver enzyme levels.^[20,21]^ From the results of this study, we believe that elevated PCT levels may be possible in instances like case 1. The two theories proposed by Tschiedel et al^[5]^ on elevated PCT levels may explain the results of case 1 as follows:

1. Hepatotoxicity may have developed without GPT level elevation as in the study on rats, due to cytokine induction after acetaminophen-induced liver cell damage.^[5]^ Furthermore, the patient was administered N-acetylcysteine 13 h after the first ingestion. This may have prevented the increase in GPT levels.
2. The second theory by Tschiedel et al,^[5]^ suggests that other organs may be responsible for elevated PCT levels.

NAPQI is known as the main cause of liver toxicity in acetaminophen intoxication.^[16,20,21]^ The involvement of other organs such as endothelial cells associated with NAPQI may have caused PCT levels to increase. However, while the difference in GPT levels between the two cases was >1000 IU/L, the differences in PCT levels was small (13 ng/mL); therefore, the two theories mentioned above may be insufficient. Further studies on PCT levels in staggered acetaminophen intoxication with a larger number of subjects are needed to draw a strong conclusion. However, the authors believed these observations in our case series and previous studies might supply the evidence that the mechanisms of PCT level elevation in patients diagnosed with acetaminophen intoxication may not be directly related to hepatic cell damage. Rather, other mechanisms such as cytokine stimuli or other organ involvement may be involved in the production of PCT in patients with acetaminophen intoxication.

The authors of this study believe that the following two studies may be useful in determining the pathophysiology of the correlation between PCT and acetaminophen levels:

1. Analysis of subtypes of PCT in patients with acetaminophen intoxication. This study would help us clarify sources of PCT that result in elevated levels. One case study has reported that that although PCT differs from gene and mRNAs expressions, it is secreted in most tissues of the human body.^[3]^ In vitro results suggested that tissues differed significantly not only in the intensity of CALC-I gene expression, but also in proportions of PCT-I, PCT-II, and CGRP mRNA produced.^[3]^ Commercially available PCT immunoluminescence assay used in most clinical studies cannot differentiate between PCT-I and PCT-II.^[3]^
2. A study that investigates the relationship between NAPQI known as a main cause of liver toxicity of acetaminophen^[16,20,21]^ and PCT.

It might provide evidence of the mechanism involved in the elevation of PCT and develop a tool to predict liver failure in acetaminophen intoxication.

In summary, we observed that PCT levels were increased in patients with acetaminophen intoxication. The significance was that the increase in PCT levels was much higher than that associated with sepsis, and levels were increased regardless of liver enzyme levels (GPT, 16 IU/L in case 1 vs 1473 IU/L in case 2) and blood acetaminophen level (<5.0 μg/mL in case 1 vs 116.9 μg/mL in case 2). The case of staggered overdose ingestion had low acetaminophen and normal liver enzyme levels. Although the mechanisms and correlation between acetaminophen intoxication and elevated PCT levels are still unclear, these observations might add to the evidence that elevated levels of PCT in patients intoxicated with acetaminophen might be associated with the possible involvement of unnoticed other organs or by cytokine stimuli from sterile inflammation resulting from hepatic damage rather than PCT secretion resulting directly from hepatic cell damage.

## Acknowledgments

We thank Dr. Hyo Jung Kang for her helpful suggestions and critical review regarding the content of the manuscript.

## Author contributions

**Conceptualization:** Jung Hwan Ahn, Gyuchong Cho.

**Data curation:** Jung Hwan Ahn, Young Suk Cho, Gyuchong Cho.

**Investigation:** Young Suk Cho.

**Methodology:** Jung Hwan Ahn, Gyuchong Cho.

**Supervision:** Young Suk Cho, Gyuchong Cho.

**Validation:** Gyuchong Cho.

**Writing – original draft:** Jung Hwan Ahn.

**Writing – review & editing:** Young Suk Cho, Gyuchong Cho.

Gyuchong Cho: 0000-0001-9228-3674.

Gyuchong Cho orcid: 0000-0001-9228-3674.
